# Decontamination of aerosolised bacteria from a pig farm environment using a pH neutral electrochemically activated solution (Ecas4 anolyte)

**DOI:** 10.1371/journal.pone.0222765

**Published:** 2019-09-25

**Authors:** Sangay Tenzin, Abiodun David Ogunniyi, Manouchehr Khazandi, Sergio Ferro, Jonathon Bartsch, Simon Crabb, Sam Abraham, Permal Deo, Darren J. Trott

**Affiliations:** 1 Australian Centre for Antimicrobial Resistance Ecology, School of Animal and Veterinary Sciences, The University of Adelaide, Roseworthy SA, Australia; 2 Ecas4 Australia Pty. Ltd., Mile End South SA, Australia; 3 Dr Barry Lloyd Pty. Ltd., Gawler SA, Australia; 4 School of Veterinary and Life Sciences, Murdoch University, Murdoch WA, Australia; 5 School of Pharmacy and Medical Sciences, University of South Australia, City East Campus, Playford P1-25, Adelaide SA, Australia; Public Health England, UNITED KINGDOM

## Abstract

An electrochemically activated solution (ECAS), generated by electrolysis of a dilute sodium chloride solution in a four-chamber electrolytic cell (Ecas4), was tested as a sanitising aerosol in eliminating bacteria from the environment of a weaning room vacated 24-48h earlier, at a continuous flow pig farm. An ultrasonic humidifier was used to fill the environment with a fog (droplets with diameters of 1–5 μm) containing 0.25 ppm of hypochlorous acid. The weaning room was fogged for 3 min at 30 min intervals during five hours of aerosol disinfection. An innovative sample treatment with propidium monoazide dye in conjunction with cyclonic air sampling was optimised and adapted for discerning live/dead bacteria in subsequent molecular quantification steps. Without fogging, total bacterial load ranged from 5.06 ± 0.04 to 5.75 ± 0.04 Log_10_ CFU/m^3^. After the first hour of fogging, a 78% total bacterial reduction was observed, which further increased to > 97% after the second hour, > 99.4% after the third and 99.8% after the fourth hour, finally resulting in a 99.99% reduction from the farm environment over five hours. Unlike the current formaldehyde spray disinfection protocol, which requires a long empty period because of its hazardous properties, this economically viable and environmentally friendly disinfection protocol may significantly lower downtime. Moreover, ECAS fogging can be easily adapted to a variety of applications, including the elimination of pathogens from livestock farm air environment for disease prevention, as well as decontamination after disease outbreaks.

## Introduction

Bacterial and/or viral respiratory diseases are a major economic cost for pork production systems. Prevalence of swine respiratory diseases are influenced by interaction of multiple factors such as the aetiological microorganisms, environmental factors of the farm such as temperature and moisture or the presence of particulate matters and ammonia, and management factors such as animal density, vector control, and disinfection strategies [1–5]. The level of production losses varies depending on the causative agents involved and their virulence. Bacterial pathogens, such as *Actinobacillus pleuropneumoniae*, may cause chronic or acute respiratory disease requiring prophylactic and/or metaphylactic antimicrobial use for adequate control [6]. Cleaning and disinfection of the animal’s environment, in conjunction with quarantine, vaccination and antibiotic treatment, constitute an important component of prevention and control of respiratory diseases [7]. In association with vaccination and the use of antibiotics, the prevention of porcine respiratory disease complex has been achieved by managing the environmental stressors through usage of disinfectants such as sodium hypochlorite, hydrogen peroxide, chlorine dioxide, formaldehyde, quaternary ammonium compounds, peroxyacetic acid and iodophors. While all these chemicals are very effective in reducing microbial load, there are limitations including differential effectiveness against a variety of bacteria [8], co-selection of antibiotic resistance [9], corrosiveness [10], concerns with skin irritation, and cross resistance to other disinfectants and antibiotics [11].

As an alternative, an electrochemically activated solution (ECAS) may provide efficacy without detrimental side effects. ECAS is a broad-spectrum bactericidal “metastable” solution containing free available chlorine (FAC) species such as hypochlorous acid and hypochlorite [12] generated from a dilute brine solution (~ 0.5% w/v). It has a disinfection activity equivalent to 80% ethanol and is superior to 0.1% chlorhexidine or 0.02% povidone iodine [13]. However, applications of ECAS produced with the original two-chamber systems have been limited due to corrosion of processing equipment [14]. Advancements in the technique for generating ECAS with slightly acidic pH (5–6.5) has allowed the use of ECAS in sanitisation of vegetables with excellent efficacy [15–17]. Furthermore, ECAS with neutral pH having excellent oxidative properties and drastically reduced corrosiveness is increasingly being employed as a sanitiser in the food industry [18, 19] and as a wound dressing product [20]. ECAS is also employed in preventing or reducing rates of infection with MRSA in post-operative infections [21, 22] in place of broad-spectrum antibiotics whose use is limited due to the development of antibiotic resistance. In addition, ECAS is being employed in disinfection and eradication of *Legionella* biofilms in hospitals and aged care facilities water systems [23, 24].

The present work was undertaken to investigate the efficacy of an electrochemically activated solution, Ecas4 anolyte, applied as a fog, in reducing the total bacterial load in a pig barn environment. In this study, we performed *in vitro* kill kinetics of Ecas4 anolyte on the swine pathogen *Actinobacillus pleuropneumoniae* (*A*. *pleuropneumoniae*) and livestock associated methicillin-resistant *Staphylococcus aureus* (MRSA) that could be transmitted through aerosols. A propidium monoazide (PMA) live/dead quantitative polymerase chain reaction (qPCR) for *A*. *pleuropneumoniae* was developed for detection and quantification. Moreover, to assess the effectiveness of the Ecas4 fogging technique in decontamination of the emptied weaning room, we optimised an air sampling technique for bacteria detection and quantification using a Coriolis cyclonic air sampler.

## Materials and methods

### In vitro kill kinetics of Ecas4 anolyte on Australian *A*. *pleuropneumoniae* and MRSA isolates

*A*. *pleuropneumoniae* serovars 1, 5, 7, 12 and 15 were obtained from Ace Laboratories (Bendigo, Victoria, Australia) and stored in culture collections at the School of Animal and Veterinary Sciences, University of Adelaide, and School of Pharmacy and Medical Sciences, University of South Australia. MRSA isolates ST30, ST93, ST398 recently isolated within a piggery environment in Australia [25] were obtained from the School of Veterinary and Life Sciences, Murdoch University. *A*. *pleuropneumoniae* ATCC 27090 and *S*. *aureus* ATCC 29213 were used as controls. *A*. *pleuropneumoniae* and MRSA isolates were originally collected by veterinarians for disease surveillance and clinical diagnosis.

*In vitro* kill kinetics were determined for each of the *A*. *pleuropneumoniae* serovars and MRSA (sequence types ST30, ST93, ST398, and *S*. *aureus* ATCC 29213) grown overnight (20–24 h) on chocolate and sheep blood agar, in 5% CO_2_ and aerobic conditions at 37 ± 1°C _,_ respectively. Subsequently, bacteria from overnight cultures were suspended in 5 mL of phosphate buffered saline (PBS) to obtain *A*_600nm_ of 0.1 (10^4^−10^5^ CFU/mL). Based on our preliminary data, where Ecas4 anolyte at lower concentrations of FAC has shown to be readily quenched by the presence of organic material in Veterinary Fastidious Medium (VFM) and cation-adjusted Mueller-Hinton (CA-MH) broth, a slightly modified Clinical and Laboratory Standards Institute [26] method was used for the kill-time assay. Briefly, PBS (1×) was used for the antimicrobial activity of various concentrations of Ecas4 anolyte (2-fold dilution from 25% to 0.49% v/v) versus contact time. A bacterial suspension (5 μL) (*A*_600nm_ = 0.1) was added to wells 2 to 12 in duplicate for all isolates and treated for various contact times, ranging from 30 s to 10 min. Well 1 contained only PBS whereas well 2 contained bacterial suspension without any Ecas4 anolyte treatment. For each contact time point, an aliquot (20 μL) from each well was plated onto chocolate and Sheep blood agar (SBA) plates for *A*. *pleuropneumoniae* and MRSA, respectively. SBA plates were incubated at 37°C for 24 h in normal environmental conditions, whereas chocolate plates were incubated at 37°C for 24 h in 5% CO_2_. Ampicillin was used as bactericidal compound with a minimum inhibitory concentration (MIC) breakpoint of 0.5–2 μg/mL as a reference for *A*. *pleuropneumoniae* ATCC 27090 and *S*. *aureus* ATCC 29213. Test validity was determined based on acceptable growth in the control well, and MIC results determined as the lowest concentration of Ecas4 anolyte that totally inhibited the growth of organisms.

### Development of live/dead real time PCR for *A*. *pleuropneumoniae* and total bacterial load detection

A previously published [27] TaqMan real-time PCR primer for detection of the *apxIVA* gene was optimised using 10-fold dilution series (from 0.005 pg/μL to 5,000 pg/μL) of *A*. *pleuropneumoniae* ATCC 27090 genomic DNA and the KAPA SYBR FAST qPCR Master Mix (Kapa Biosystems, Inc., Cape Town, South Africa). The reaction mix consisted of a 6 μL aliquot of master enzyme reaction super mix, various volumes of sense and antisense primers to achieve 0.25 μM, 0.5 μM, 0.75 μM, 1 μM or 2 μM concentrations, and nuclease free water to obtain 10 μL including 1 μL of DNA template. The real time PCR run parameters were set up with initial denaturing step at 95°C for 5 min followed by 40 cycles of denaturation at 95°C for 15 s, primer annealing and extension at 60°C for 30 s, where DNA amplification was acquired on green channel, with a final extension step at 40°C for 60 s (Bio Molecular Systems, NSW 2011, Australia).The specificity of the *A*. *pleuropneumoniae* optimised primers was tested against DNA from the following bacterial strains: *A*. *lignieressi*, *A*. *suis*, *Mannheimia haemolytica*, *Pasteurella multocida*, *S*. *suis*, *Bordetella broncheseptica*, and *Haemophilus parasuis*.

Similarly, a set of universal primers for amplification of bacterial 16S rDNA [28] was optimised for detection and quantification of recoverable bacterial load by qPCR with a quantification curve generated using 10-fold serial dilutions up to 1:10,000 dilutions (from 0.012 pg/μL to 12,000 pg/μL) of *E*. *coli* ATCC 35218 gDNA. A 10 μL total reaction volume containing 6 μL of master enzyme reaction super mix from KAPPA fast qPCR SYBR green kit, 0.25 μM, 0.5 μM, 0.75 μM and 1 μM each of forward and reverse primers, varying volumes of nuclease free water and 1 μL DNA template per sample were used to perform qPCR. The qPCR amplification conditions mentioned above were used.

Since qPCR detects all dsDNA sequences specific to the primers used, samples were treated with propidium monoazide (PMA) (a photoreactive membrane-impermeable dye that selectively penetrates bacterial cells with compromised membranes considered dead and binds covalently to dsDNA), prior to DNA extraction for qPCR described above. To optimise the detection and differentiation of live/dead *A*. *pleuropneumoniae* as well as the recoverable bacterial load in air samples, 24h growth *A*. *pleuropneumoniae* and *E*. *coli* were suspended in PBS to an *A*_600nm_ of 0.58. Bacterial suspension (50 μL) was added to Ecas4 anolyte (5 mL, 0, 5% and 10% diluted in milliQ water) and incubated at room temperature for 5 min. After incubation, aliquots (100 μL) were plated onto either chocolate agar for *A*. *pleuropneumoniae* or SBA for *E*. *coli* and incubated as detailed above.

Additionally, aliquots (4 × 500 μL) of *A*. *pleuropneumoniae* and *E*. *coli* suspension in PBS were heated at 85°C for 10 min to kill cells by compromising their bacterial membrane integrity and used as additional bacterial death controls. All bacterial suspensions (Ecas4 anolyte and heat treated samples) were further treated with 6.25, 12.5, 25 and 50 μL of 2 mM PMA to obtain final concentrations of 25, 50, 75 and 100 μM per reaction, vortexed briefly, and incubated for 15 min at room temperature in the dark. After incubation, samples were exposed to light using a PMA-Lite LED photolysis device (Biotium, Inc., Fremont, CA 94538, USA) for 15 min to allow PMA photoactivation. An untreated PMA bacterial suspension from each treatment was used as control. All samples were centrifuged at 12,400 × *g* for 10 min, discarding the supernatant and resuspending the pellet in 70 μL of nuclease free water.

For DNA extraction, 280 μL of lysis buffer were added to each sample, vortexed and incubated for 10 min. Samples were then pipetted into silicone membrane filter tubes and centrifuged at 9,500 × *g* for 60 s. The supernatant was discarded and pre-wash buffer (200 μL) was added to all tubes, mixed and centrifuged (1 min at 9,500 × *g*), before discarding the supernatant again. The procedure was repeated with wash buffer (500 μL). Elution buffer (35 μL) was added to all the tubes and then samples were incubated for 3 min at room temperature before centrifuging at 12,400 × *g* for 60 s. DNA concentration was measured by using a DeNovix DS-11+ spectrophotometer. qPCR runs to determine the PMA concentration required for the discernment of live and killed bacteria were performed using the optimised parameters mentioned above.

### Air sampling from the farm–Optimisation study

**Fig 1:**
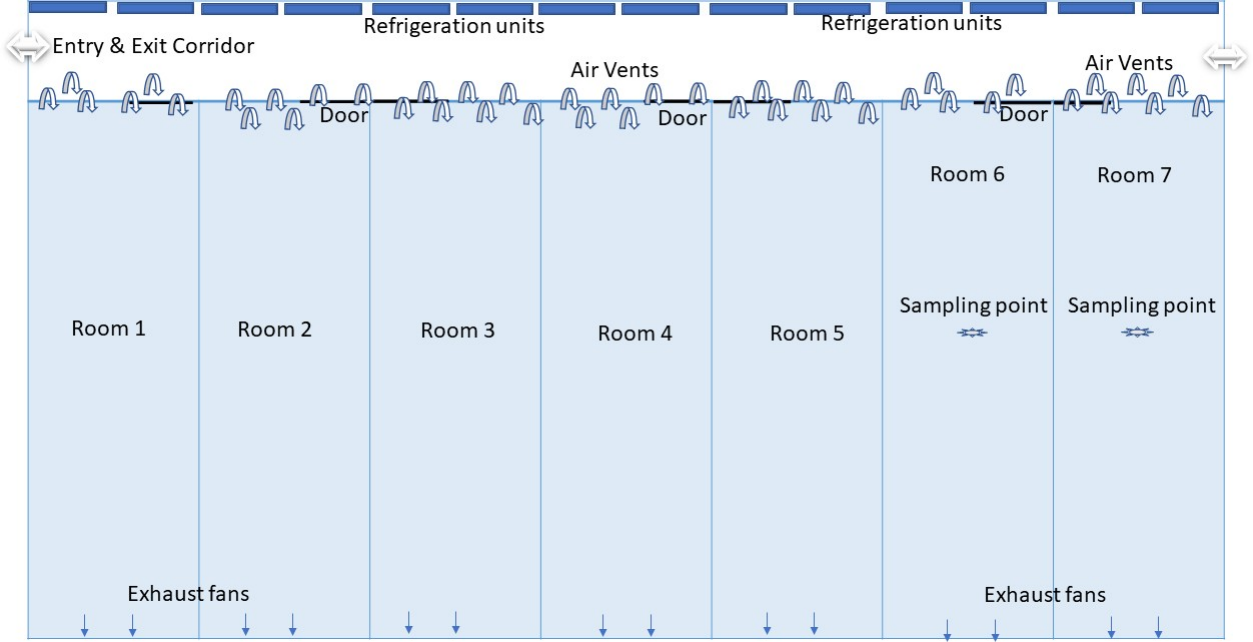
Scheme of the weaning shed with seven weaning rooms and locations of exhaust fans, air vents, refrigeration units and sampling points.

The air sampling procedure was optimised in a commercial piggery in South Australia; consent was obtained from farm management and all experiments were carried out under the supervision of the farm manager. The piggery shed contained seven weaning rooms of equal size (145 m^3^) with partially slated floors. Each room was ventilated by means of two automatically controlled exhaust fans with temperature set at 23°C (Fig 1). Air coolers were mounted on the side walls of the entrance and exit corridor of the shed, and the cool air sucked into the individual rooms through the air vents located in the upper part of the walls located opposite to exhaust fans (Fig 1). Humidity ranged from 24 to 58%. The air vents were designed to guide the incoming air downwards, and the opening of air vents regulated by the differential pressure created in the room by two exhausts fans operating in synchrony.

Air samples from an empty weaning room were collected from the centre of the room (1.15 m height above the floor level–Fig 1) using a Coriolis cyclonic air sampler (Bertin technologies, Montigny-le-Bretonneux—France). Various collection volumes (5, 7.5, 10, and 12.5 mL) of VFM for different collection times (2, 3, 4, 5, and 6 min) with air sample volumes of 250 and 300 L/min were used during the optimisation trial. The volume of VFM and the collection time were optimised in order to obtain the best detection and quantification of *A*. *pleuropneumoniae* and other bacteria. Collected samples were immediately refrigerated on ice, transported to the laboratory and processed for total recoverable bacterial counts as well as live/dead qPCR.

Sample aliquots (100 μL) in VFM were used for the preparation of serial dilutions ranging from 10 to 10^−6^ in 0.1% peptone water, inoculated onto plate count agar plates and incubated overnight at 37 ± 1°C in aerobic condition to estimate bacterial counts. The results obtained in CFU/mL were then converted into CFU/m^3^.

### Air sampling in the weaning room with and without Ecas4 anolyte fogging

Prior to Ecas4 anolyte fogging experiments, the total recoverable bacterial load in the empty weaning room (within 24–48 h of the animal transfer) was assessed by collecting samples of farm air, using the Coriolis air sampler and the optimised parameters for air collection (250 L/min for 3 min, in 10 mL of sterile VFM). Five air samplings were undertaken over five hours with a one-hour interval. After each sampling, the sampler was purged with 0.1% hydrogen peroxide (H_2_O_2_) before the next sample collection. Samples were transported on ice to the laboratory as described above. Aliquots (1 mL) of all the samples in VFM were centrifuged at 12,400 × *g* for 10 min, the supernatant discarded and pellet resuspended in 100 μL of nuclease free water. Then, each sample was treated with propidium monoazide (PMA) dye (Biotium Inc. CA 945, USA) at 100 μM concentration per sample. PMA binding, DNA extraction and live/dead qPCR for detection and quantification of *A*. *pleuropneumoniae* and total bacterial load were carried out as mentioned earlier. In addition, 100 μL aliquots of samples in VFM were also used to prepare serial dilutions ranging from 10 to 10^−6^ in 0.1% peptone water, inoculated onto plate count agar plates and incubated overnight at 37 ± 1°C in aerobic conditions to estimate total recoverable bacterial counts.

For fogging, a 50% v/v Ecas4 anolyte containing 150 ppm of FAC was used to generate a fog containing 0.25 ppm (0.75 mg/m^3^) of FAC. Within 24–48 h of the animal transfer, the room was fogged for 3 min at 30-min intervals for 5 h, using an ultrasonic humidifier (Aqua Aircon M18K, Shang-E International Resources Development Corporation, Taiwan) that produces a mist with droplets having a diameter between 1 and 5 μm. Air samples were collected before fogging (sample 0) and then every hour for five hours (samples 1–5) during fogging using the Coriolis air sampler at a rate of 250 L/min for 3 min and with 10 mL of sterile VFM. Samples were collected and analysed in duplicate and the experiment trialled independently over 2 days. Samples were transported to the laboratory on ice to undertake total recoverable bacterial counts as well as live/dead qPCR as described above.

## Results

### In vitro kill kinetics of Ecas4 anolyte on *A*. *pleuropneumoniae* and MRSA

The *in vitro* antimicrobial susceptibility testing of Ecas4 anolyte against field strains of *A*. *pleuropneumoniae* (representing major serotypes 1, 5, 7, 12, 15, and ATCC 27090) and MRSA (representing sequence type 398, the main livestock-associated MRSA and the important human-disease associated clone ST93) recently isolated from Australian pigs, showed that very low concentrations of Ecas4 anolyte in water are effective in killing both *A*. *pleuropneumoniae* (0.39% v/v, 1.17 μg/mL) and MRSA isolates (0.78% v/v, 2.34 μg/mL) within 30 s of exposure. Ampicillin at 2.0 μg/mL was effective in killing 99.99% of both *A*. *pleruopneumoniae* and *S*. *aureus* ATCC strains.

### Development of live/dead real time PCR for *A*. *pleuropneumoniae* and total bacterial load estimation

**Fig 2:**
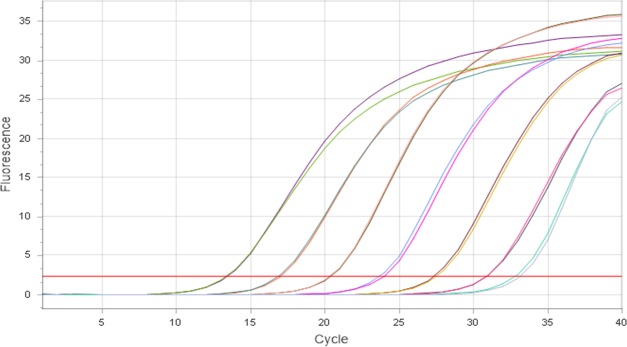
Amplification curves of 10-fold serial dilutions of *A*. *pleuropneumoniae* ATCC DNA (from 0.05 to 5,000 pg).

**Fig 3:**
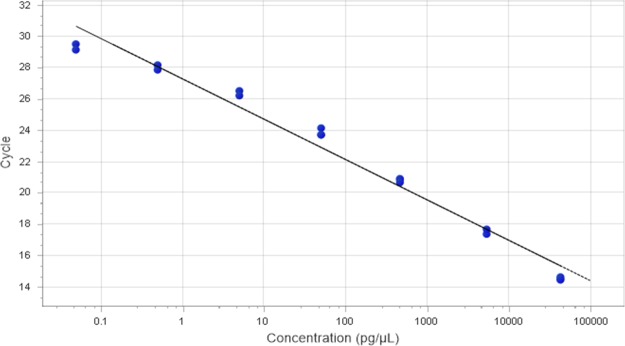
Calibration curve of serial dilutions of *A*. *pleuropneumoniae* ATCC DNA.

Reverse and forward primers for *A*. *pleuropneumoniae apxIVA* gene at concentrations of 1.0 μM in a reaction volume of 10 μL could detect and quantify as little as 0.05 pg/μL of *A*. *pleuropneumoniae* ATCC 27090 and other *A*. *pleuropneumoniae* serovars. Primer pairs of lower concentration (0.25 μM to 0.75 μM) could not detect *A*. *pleuropneumoniae* at a concentration of 0.05 pg/μL. However, a primer pair concentration of 2.0 μM could quantify 0.05 pg of *A*. *pleuropneumoniae* gDNA, although it caused primer-dimer formation at around the 30^th^ quantification cycle (Cq). Quantification curves of serial dilutions of *A*. *pleuropneumoniae* ATCC 27090 from 5,000 ng to 0.05 pg per reaction and their corresponding Cq values for *A*. *pleuropneumoniae* ATCC 27090 DNA serial dilutions are shown in Fig 2 and Table 1; the corresponding calibration curve is shown in Fig 3. The specificity of *apxIVA* gene primers was high as no nonspecific amplification occurred with other tested porcine nasal bacteria.

**Table 1:** Cq values of *A*. *pleuropneumoniae (A*. *pp)* ATCC 20790 DNA serial dilutions.

Well	Sample	DNA conc. (pg/μL)	Cq	R^2^
1	*A*. *pp* ATCC 1:1	5,000	13.41	0.99
2	*A*. *pp* ATCC 1:1	5,000	13.37	0.99
3	*A*. *pp* ATCC 1:10	500	17.04	0.99
4	*A*. *pp* ATCC 1:10	500	16.96	0.99
5	*A*. *pp* ATCC 1:100	50	20.37	0.99
6	*A*. *pp* ATCC 1:100	50	20.33	0.99
7	*A*. *pp* ATCC 1:1,000	5.0	23.69	0.99
8	*A*. *pp* ATCC 1:1,000	5.0	23.97	0.99
9	*A*. *pp* ATCC 1:10,000	0.5	27.35	0.99
10	*A*. *pp* ATCC 1:10,000	0.5	27.54	0.99
11	*A*. *pp* ATCC 1:100,000	0.05	30.92	0.99
12	*A*. *pp* ATCC 1:100,000	0.05	30.88	0.99

**Fig 4:**
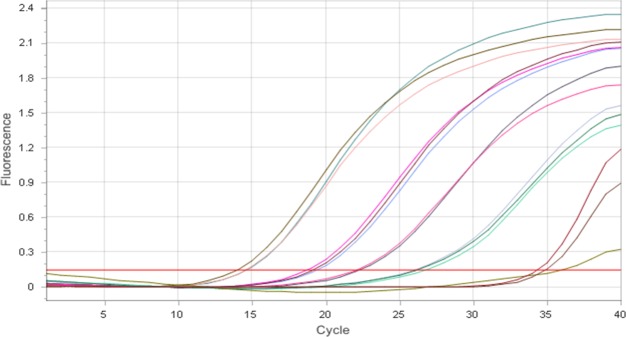
Amplification curves of 10-fold serial dilutions of *E*. *coli* DNA.

Regarding the 16S rDNA universal bacterial gene primers, 0.5 nM and 0.75 nM of forward and reverse primers in a final volume of 10 μL were able to detect *E*. *coli* DNA concentrations as low as 1.2 pg/μL. The qPCR reaction efficiency of the 0.75 nM primer set is 1.17% and the correlation coefficient is 0.99 for the total bacterial qPCR. 1.0 nM primer set caused primer dimerization at the 32^nd^ quantification cycle (Cq). The quantification curve of 10-fold serial dilutions of *E*. *coli* and corresponding Cq values are presented in Table 2 and Fig 4, respectively.

**Table 2:** Cq values of *E*. *coli* DNA serial dilutions.

Sl. No.	Sample	*E*. *coli* DNA conc. (pg/μL)	Cq	R^2^
1	*E*. *coli* 1:10	4,100	15.14	0.99
2	*E*. *coli* 1:10	4,100	14.71	0.99
3	*E*. *coli* 1:10	4,100	15.24	0.99
4	*E*. *coli* 1;100	151	19.91	0.99
5	*E*. *coli* 1;100	151	19.33	0.99
6	*E*. *coli* 1;100	151	19.59	0.99
7	*E*. *coli* 1:1,000	15	23.42	0.99
8	*E*. *coli* 1:1,000	15	22.52	0.99
9	*E*. *coli* 1:1,000	15	22.61	0.99
10	*E*. *coli* 1:10,000	1.2	25.77	0.99
11	*E*. *coli* 1:10,000	1.2	25.17	0.99
12	*E*. *coli* 1:10,000	1.2	25.57	0.99

**Fig 5:**
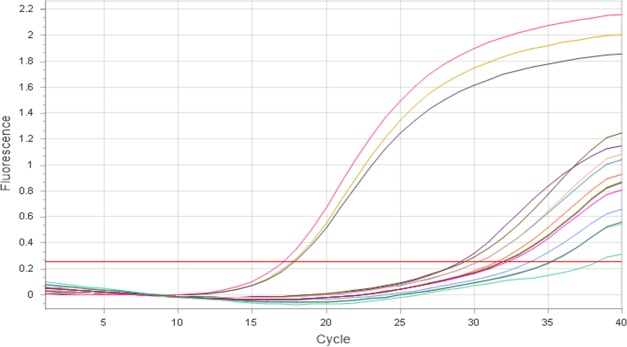
Amplification curve of *A*. *pleuropneumoniae* ATCC 27090 DNA: (1) from cells killed by boiling (PMA treated– 27.5 Cq); (2, 3) from cells killed with Ecas4 anolyte at 5% (no PMA treatment– 26.0 Cq) and 10% (no PMA treatment– 30.4 Cq); (4) from a viable (live) control (15.3 Cq). qPCR was performed with 1.0 μM *apxIVA* gene primer.

**Fig 6:**
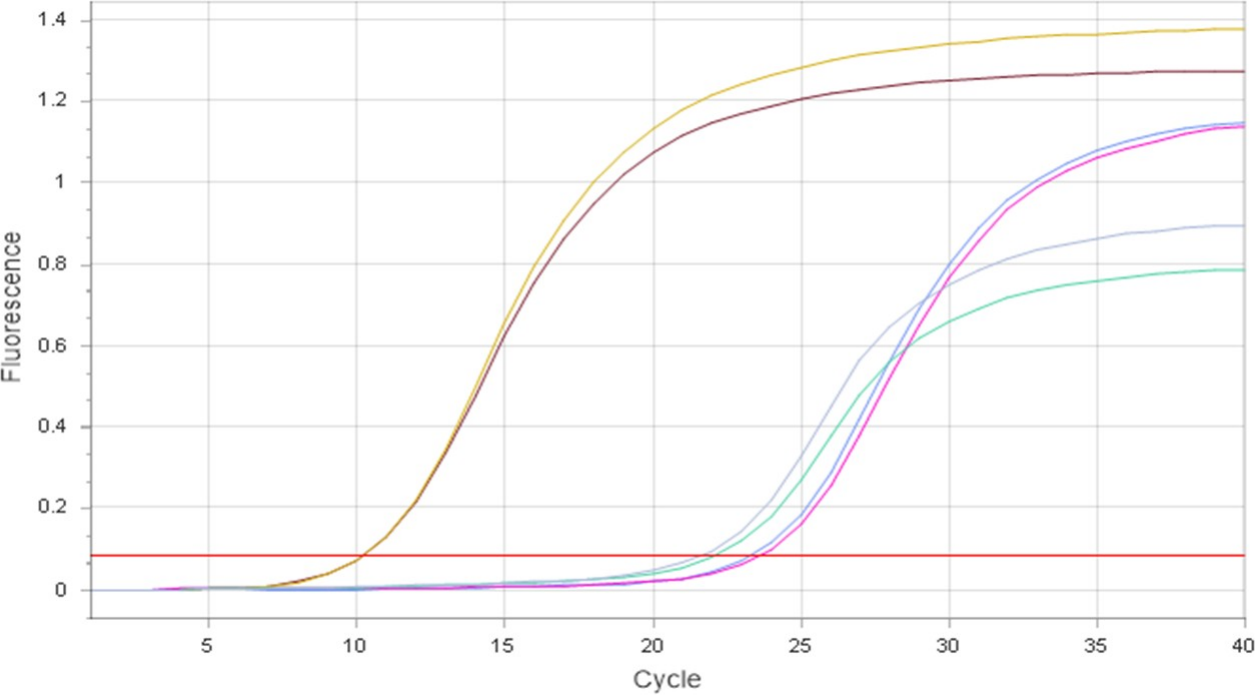
Amplification curves of *E*. *coli* DNA: (1) from viable (live) cells (10.5 Cq); (2) from cells killed with Ecas4 at 5%, without PMA treatment (22.0 Cq); (3) from dead *E*. *coli* control treated with 50 μM PMA (23.7 Cq–Aliquot of 500 μL killed by boiling at 85°C for 15 min). All qPCR tests were performed using universal bacterial primers at 0.75 nM.

Amplification of *A*. *pleuropneumoniae* ATCC 20790 killed with 5% and 10% Ecas4 anolyte, dead control (500 μL of aliquot killed by boiling at 85°C for 15 min and treated with 50 μM PMA) and viable (live) controls quantified employing *apxIVA* gene primers at 1.0 μM concentrations are shown in Fig 5. Cq values of viable control and dead control were 15.3 and 27.7, respectively; Cq values of amplified DNA of viable (live) *E*. *coli* (10.5), dead *E*. *coli* control (23.5—treated with 50 μM PMA) and 5% ECAS-killed (22.0) samples quantified using universal bacterial primers at 0.75 nM are shown in Fig 6. The treatment of dead control samples with PMA 50 μM resulted in more than 10,000 fold reduction in DNA amplification in comparison to that of the viable (live) controls in both *A*. *pleuropneumoniae* and *E*. *coli*. With 75 μM and 100 μM PMA, Cq value differences between the dead and viable controls observed were similar to those of the treatment with PMA 50 μM. However, with 25 μM PMA no differentiation of live and dead control for both bacteria were recorded.

### Detection of *A*. *pleuropneumoniae* and total bacterial load in air samples–Optimisation study

The optimised parameters for the collection of the farm’s environmental air samples were 10 mL of VFM as the collection liquid and a pre-set collection air volume of 250 L/min for 3 min. These parameters allowed detection of *A*. *pleuropneumoniae* at 200 pg (5.1×10^6^ GU) in 2 mL of VFM and total recoverable bacterial gDNA of 1,200 pg (1.5×10^8^ GU) in 2 mL of VFM. However, the quantity of detectable *A*. *pleuropneumoniae* increased to about 1,600 pg (2.1×10^8^ GU) in 2 mL when samples were incubated in VFM overnight in 5% CO_2_. Total recoverable bacterial load, in terms of genomic units of DNA present, also increased to 3.6×10^9^ GU after overnight incubation in aerobic conditions.

### Bacterial quantification in the air sampled in the weaning room before Ecas4 anolyte fogging

Total recoverable bacteria loads and bacterial DNA concentrations quantified in an empty weaning shed prior to fogging with Ecas4 anolyte are presented in Table 3. The total recoverable bacterial load ranged from 5.06 ± 0.04 Log_10_ CFU/m^3^ to 5.75 ± 0.04 Log_10_ CFU/m^3^. Bacterial DNA concentrations evaluated using real-time qPCR ranged from 2,021 ± 146 pg/μL to 3,218 ± 117 pg/μL. Interestingly, *A*. *pleuropneumoniae* was not detected in any of the collected air samples.

**Table 3:** Total bacteria and DNA concentration in the air sampled in an empty weaning room over five hours. Data reported as mean ± standard error of mean (SEM), number of replicates (n) = 2.

Samples	Total bacteria (Log_10_ CFU/m^3^)	DNA concentration (pg/μL)
First hour	5.74 ± 0.01	2,021 ± 146
Second hour	5.07 ± 0.02	2,412 ± 159
Third hour	5.06 ± 0.04	2,492 ± 17
Fourth hour	5.32 ± 0.01	3,046 ± 49
Fifth hour	5.75 ± 0.04	3,218 ± 117

### Bacterial quantification in the air sampled in the weaning room during Ecas4 anolyte fogging

*A*. *pleuropneumoniae* could not be detected using SYBR green-based real time qPCR for detection of a*pxIVA* gene in DNA from samples collected from the weaning shed and incubated overnight in VFM at 37 ± 1°C in 5% CO_2_.

**Fig 7:**
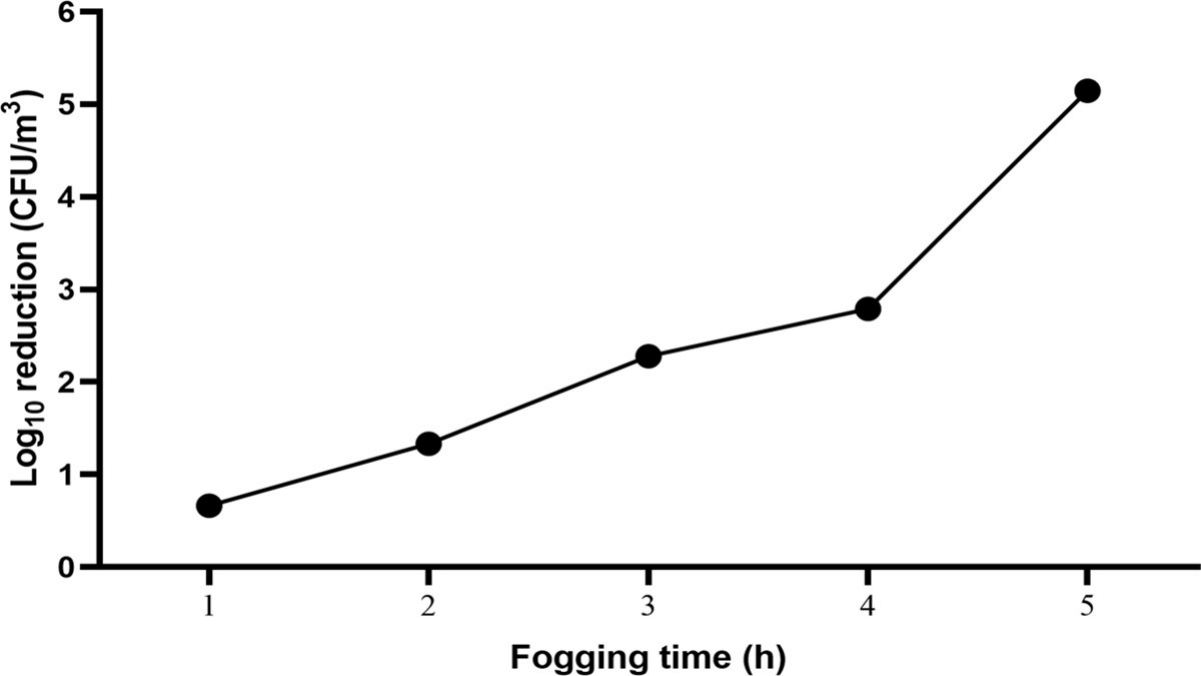
Reduction of bacterial load (Log_10_ CFU/m^3^) over time enumerated using plate count technique.

**Fig 8:**
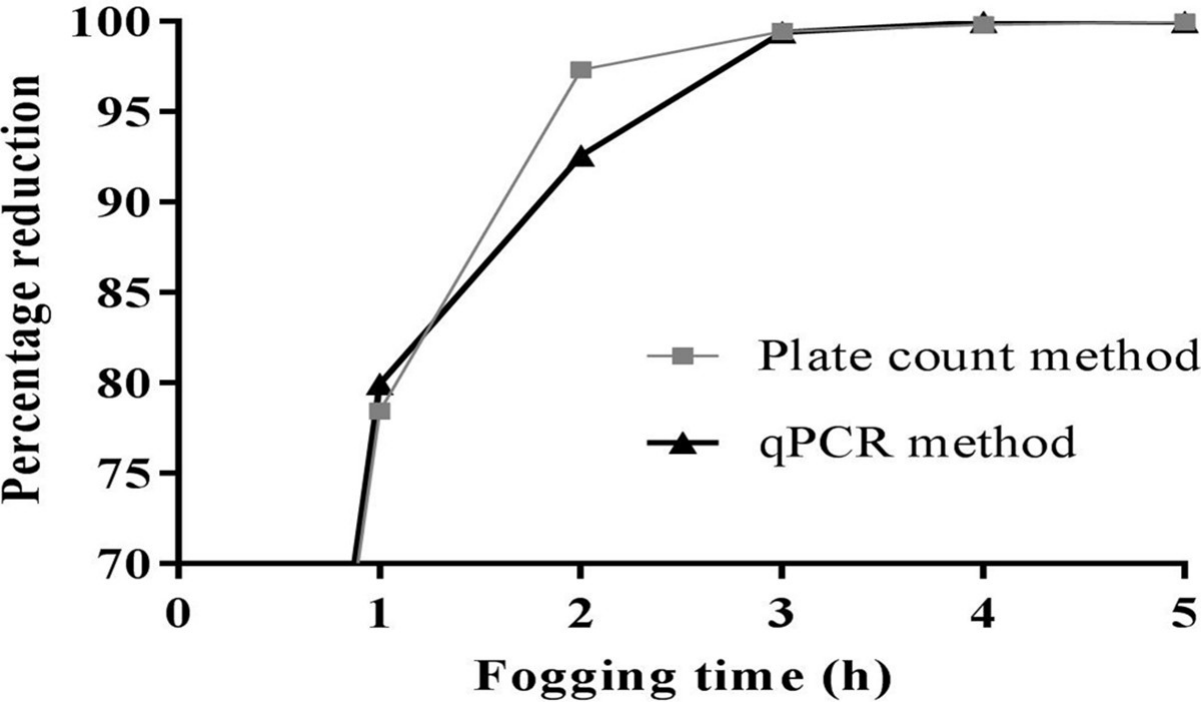
Percentage of killed bacteria enumerated using plate count and quantitative PCR methods.

Total recoverable bacterial load was 5.77 ± 0.01 Log_10_ CFU/m^3^ in the weaning room environment at the commencement of the experiment. Bacterial counts enumerated on a plate count agar and converted into CFU/m^3^ of air provided a 0.67 Log_10_ reduction in total recoverable bacterial count after the first hour of fogging, which increased to 5.12 Log_10_ after 5 hours (Fig 7). A total bacterial reduction amounting to 99.998% (Fig 8) was estimated after Ecas4 anolyte fogging (Table 4).

**Table 4:** Total bacteria and Log_10_ reduction on air samples collected from an empty weaning room during Ecas4 anolyte fogging. Data reported as mean ± SEM, n = 4.

Samples	Total Bacteria (Log_10_ CFU/m^3^)	Log_10_ reduction	Percent reduction
Before fogging (0)	5.77 ± 0.01		
1 h after fogging	5.10 ± 0.02	0.67	78.326
2 h after fogging	4.43 ± 0.05	1.34	97.361
3 h after fogging	3.49 ± 0.07	2.28	99.459
4 h after fogging	2.98 ± 0.03	2.79	99.837
5 h after fogging	0.65 ± 0.07	5.12	99.998

Real time live/dead qPCR for detection and quantification of total bacterial DNA before and after the various decontamination time points showed a 1.13 Log_10_ reduction during the first 2 h of fogging, the reduction further increased to 4.79 Log_10_ in DNA concentration (pg/μL) after 5 h of fogging (Table 5). Total bacterial load reduction was 99.998% (Fig 8) after 5 h of Ecas4 fogging at 0.25 ppm of FAC in the air.

**Table 5:** Total bacterial load (data reported as mean ± SEM, n = 2), Log_10_ and percent reductions before and after Ecas4 anolyte fogging quantified by qPCR using universal bacterial primers and live/dead differentiation.

Samples	DNA concentration (Log_10_ pg/μL)	Log_10_ reduction	Percent reduction
Before fogging (0)	2430 ± 286		
1 hour after fogging	489 ± 205	0.72	80.004
2 hours after fogging	193 ± 18	1.13	92.637
3 hours after fogging	14 ± 3	2.24	99.426
4 hours after fogging	0.051 ± 0.004	4.62	99.998
5 hours after fogging	0.039 ± 0.002	4.79	99.998

## Discussion

Various forms of electrolysed oxidising (EO) waters are becoming increasingly popular as effective and safer disinfection/sanitation options in healthcare settings and in the food industry [29]. An EO water with neutral pH, such as the Ecas4 anolyte, provides an additional level of safety (since there is no dissolved chlorine gas, the solution is not corrosive), without compromising its effectiveness. As EO water is expected to kill bacteria by breaking down covalent bonds in proteins through oxidative reactions [30], we found that a lower concentration of Ecas4 anolyte was required to kill *A*. *pleuropneumoniae* (Gram-negative bacteria, with a single peptidoglycan layer in the cell membrane) compared to the slightly higher concentrations needed to kill MRSA (Gram-positive bacteria with a peptidoglycan multilayer).

*A*. *pleuropneumoniae* primers and reaction optimum conditions were determined to detect *A*. *pleuropneumoniae* DNA concentrations as low as 0.05 pg/μL in 10 μL total reaction volume, with 100% specificity when tested against other porcine nasal bacteria. For universal bacterial primers, optimal conditions were determined by exploiting their increased reproducibility and increased reaction efficiency, which allowed quantification of as low as 1.2 pg/μL of gDNA of *E*. *coli* without primer-dimer formation before 35.0 quantification cycles.

The use of a Coriolis cyclonic air sampler was optimised to detect and quantify *A*. *pleuropneumoniae* and total bacterial load in the farm’s environment. It showed excellent repeatability for collection volumes between 500 and 1,500 L providing plate counts per cubic meter of air comparable to those of a conventional plate impactor system (Millipore M air T) [31] and higher efficiency than the AGI-impinger in a poultry farm study [32]. As in previous studies [31–32], the air sampling protocol adopted in this study proved successful in detecting very low levels of *A*. *pleuropneumoniae* in the growing/finishing and weaning sheds with pigs present, during the optimisation steps; however, no *A*. *pleuropneumoniae* was isolated from the empty weaning rooms during the fogging experiments. Recoverable bacterial counts from swine ambient air samples can vary depending on farm production type and management systems, with a total recoverable bacterial load ranging from 3.4 to 5.9 Log_10_ CFU/m^3^ in studies to date [33, 34]. The maximum recoverable bacterial load quantified in the empty weaning shed in this study was equal to 5.75 ± 0.04 Log_10_ CFU/m^3^. This relatively high bacterial load in an empty weaning room could be due to the fact that the Coriolis sampler captures bacteria with greater efficiency than the conventional impinger technique [32], but it may also reflect the high bacterial load on this particular continuous flow farm.

The initial bacterial reduction of 0.67 Log_10_ CFU/m^3^ after fogging for one hour is comparable to that achieved in our previous study, where Ecas4 anolyte at 45 ppm FAC wash sanitisation of fish fillets achieved reduction of 0.5 Log_10_ [18]. Likewise, the 1.34 Log_10_ CFU/m^3^ reduction after two hours of fogging was superior to washing fish fillets with 150 ppm FAC (1.0 Log_10_) [18]. The different effectiveness observed between the two fish fillet wash experiments and the two fogging time points could be attributed to the presence of organic matter quenching the anolyte active components, since even Ecas4 with low FAC (0.5 ppm) was able to totally inactivate bacterial cells (5 Log_10_) in the absence of organic material. The high disinfection potential of Ecas4 anolyte may be attributed to the high oxidation-reduction potential of the solution (> 1100 mV), which contains powerful oxidants that sequester electrons from the bacterial structure, damage the cell membrane and inactivate intracellular proteins, lipids and nucleic acids, making the bacterial cells non-functional [29]. The efficacy may also have been improved by applying the ECAS as a fog, as the tiny mist particles containing aerosolised oxidative moieties bind more efficiently to the bacterial surface causing more rapid cell lysis.

Increased effectiveness of aerosol disinfection by fogging was observed when pedestal fans were used to distribute the fog more evenly and rapidly in a fogging experiment [35] and this could therefore be an important factor contributing to the high efficacy in reducing bacterial load. A similar effect could be attributed to the design of the airflow in the weaning shed, whereby the airflow enters the shed on one side and is sucked out by an exhaust fan located on the opposite side of the room (Fig 1), thus allowing a rapid distribution of the fog. However, the effectiveness of the aerosol disinfection protocol might also have been influenced by the size and design of the pig farm. The Ecas4 fog at the current FAC content (0.75 mg/m^3^) was able to rapidly disinfect the air of the farm’s environment in a small closed shed with uniform air circulation. However, in larger pig sheds that do not have uniform air circulation, a longer fogging time may be needed to inactivate the greater bacterial load produced by more animals.

The live/dead qPCR total bacterial load determination optimised in this work was able to provide data on the killing of bacteria (Fig 8), similar to those obtained through conventional enumeration methods, thus validating the ability of the treatment with PMA dye to quantitatively differentiate alive and dead bacteria; however, the concentration of PMA required for treating the samples had to be adjusted to the specific collection media used. In addition, qPCR data from *A*. *pleuropneumoniae* and *E*. *coli* killed with 5% and 10% Ecas4 anolyte without PMA treatment provided Cq values similar to those of the bacteria killed by boiling at 85°C for 15 min and treated with PMA 50 μM (Figs 5 & 6), i.e. a 10,000-fold reduction in DNA amplification compared to that from viable control bacteria, suggesting that no “viable but nonculturable” (VBNC) cells are induced during the treatment, as PMA is a membrane impairment dye used for VBNC determination in conjunction with pre-mRNA reverse transcription qPCR [36, 37]. However, the lack of VBNC induction by the Ecas4 anolyte should be verified in tandem using a further viability assay, such as the 16S ribosomal RNA expression or demonstration of no metabolic activity.

Since the Ecas4 anolyte is biodegradable, certified “organic” and requires extremely short downtime during the fogging period, it could potentially lead to a reduced use of prophylactic antibiotics for the prevention of porcine respiratory disease complex by minimising the transmission of respiratory pathogens such as *A*. *pleuropneumoniae* between carrier and naïve animals, and thus minimising the risk of development of antibiotic resistance, without the problems associated with the use of other chemicals such as formaldehyde and H_2_O_2_. Moreover, the levels of chlorine in the farm’s environment at 0.25 ppm (0.75 mg/m^3^ of hypochlorous acid) would fall within the short-term exposure limit (0.5 ppm) and the time-weighted average limit (1.0 ppm) prescribed by the Safe Work Australia standards [38]. From an economical point of view, the reduction of the environmental bacterial load should help by reducing medication costs and improving food conversion and growth rate, since an increase in the concentration of bacteria in the air is a main factor in the decline of the growth rate of pigs in a high-density stocking environment, leading to high incidence of pleurisy and pneumonia [39, 40].

In conclusion, proof-of-concept data on Ecas4 anolyte as a cost-effective air decontamination agent were obtained when the anolyte was administered as a fog to prevent the transmission of bacterial pathogens. This protocol can now be adapted to a model implementable in various farm settings for the control and prevention of the transmission of aerosolised pathogenic bacteria.

## Supporting information

S1 Fig Melting curves of *Actinobacillus pleuropneumoniae* (App) DNA for qPCR performed with *apxIVA* gene primers. a. App ATCC viable control (V)–no kill b. App ATCC killed by boiling (PMA treated- D). c. Ecas4 5% killed (5EAppATCC) without PMA treatment. d. 10% Ecas4 killed (E10App ATCC)- no PMA treatment and e. NTC- no template control. 5% Ecas4–15 ppm free available chlorine (FAC), 10% Ecas4–30 ppm FAC.
(TIF)

S2 Fig Melting curves of *E*. *coli* DNA for qPCR performed using universal bacterial primers. a. E. coli ATCC viable control (V1)–no kill b. E. coli ATCC killed by boiling (PMA treated- D1) c. Ecas4 5% killed (E5 E. coli ATCC) without PMA treatment. d. 10% Ecas4 killed (E10E. coli ATCC)- no PMA treatment and e. NTC- no template control. 5% Ecas4–15 ppm free available chlorine (FAC), 10% Ecas4–30 ppm FAC.
(TIF)
