# Supplementary figures and images for: Decontamination of aerosolised bacteria from a pig farm environment using a pH neutral electrochemically activated solution (Ecas4 anolyte)
Source: PLoS One. 2019 Sep 25;14(9):e0222765. doi: 10.1371/journal.pone.0222765 (PMC6760800; doi:10.1371/journal.pone.0222765)

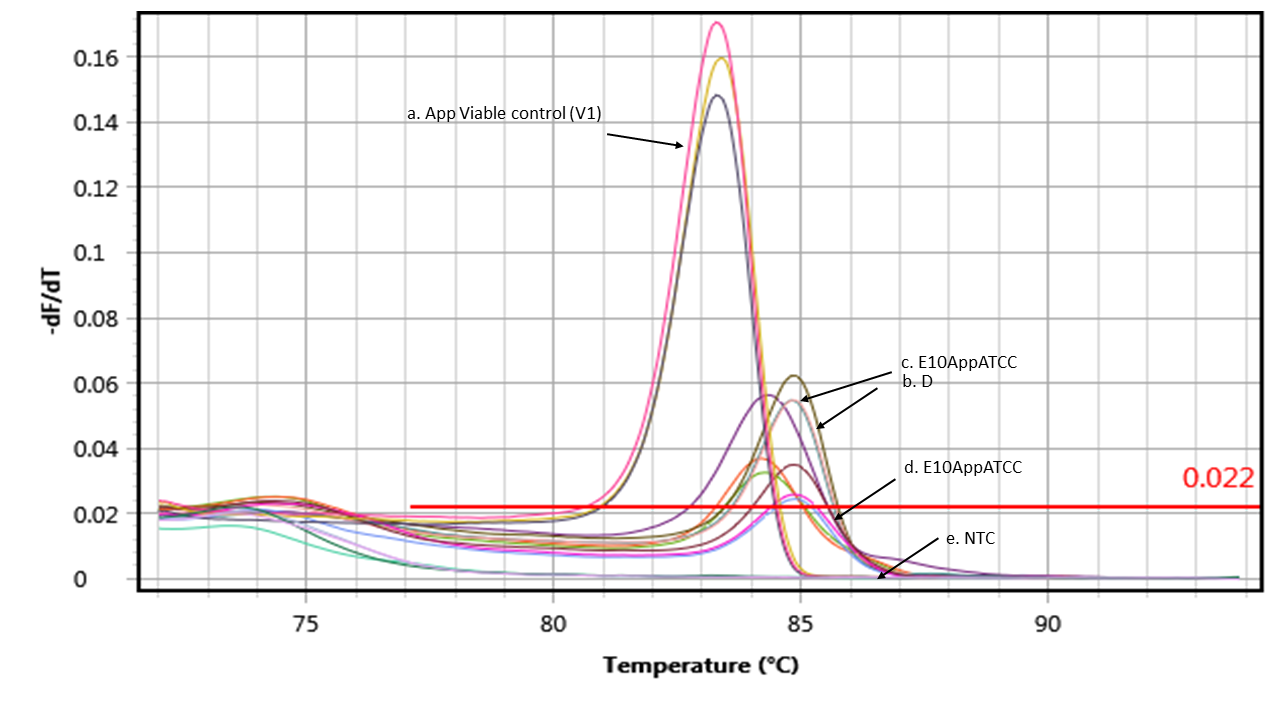

Supplement: S1 Fig — a. App ATCC viable control (V)–no kill b. App ATCC killed by boiling (PMA treated- D). c. Ecas4 5% killed (5EAppATCC) without PMA treatment. d. 10% Ecas4 killed (E10App ATCC)- no PMA treatment and e. NTC- no template control. 5% Ecas4–15 ppm free available chlorine (FAC), 10% Ecas4–30 ppm FAC. (TIF) [file pone.0222765.s001.tif]

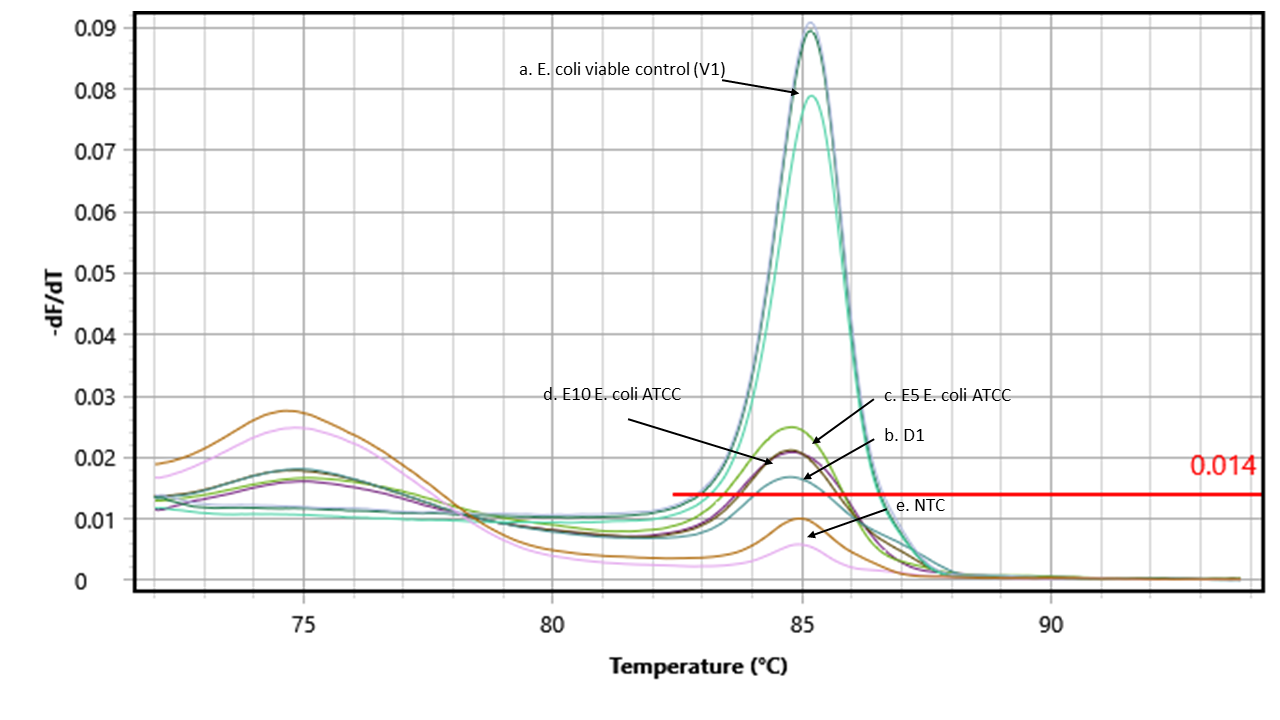

Supplement: S2 Fig — a. E. coli ATCC viable control (V1)–no kill b. E. coli ATCC killed by boiling (PMA treated- D1) c. Ecas4 5% killed (E5 E. coli ATCC) without PMA treatment. d. 10% Ecas4 killed (E10E. coli ATCC)- no PMA treatment and e. NTC- no template control. 5% Ecas4–15 ppm free available chlorine (FAC), 10% Ecas4–30 ppm FAC. (TIF) [file pone.0222765.s002.tif]
